# Mechanical Resistance and Tissue Structure of Claw Denticles of Various Sizes in the Mud Crab, *Scylla serrata*

**DOI:** 10.3390/ma16114114

**Published:** 2023-05-31

**Authors:** Tadanobu Inoue, Yuka Hara, Koji Nakazato

**Affiliations:** National Institute for Materials Science, 1-2-1, Sengen, Tsukuba 305-0047, Japan; hara.yuka@nims.go.jp (Y.H.); nakazato.koji@nims.go.jp (K.N.)

**Keywords:** biomineralization, cuticle bulge, microstructure, chemical composition, nanoindentation, abrasion resistance, hardness

## Abstract

Decapod crustaceans have tooth-like denticles on their claw fingers, which come into direct contact with predators and prey. Since the denticles are subject to more frequent and intense stress than other parts of the exoskeleton, they must be especially resistant to wear and abrasion. We clarified the mechanical resistance and tissue structure of the denticles arranged in a line on the fixed finger of the mud crab, which has huge claws. The denticles of the mud crab are small at the fingertip and become larger closer to the palm. The denticles have a twisted-plywood-pattern structure stacked parallel to the surface regardless of size, but the abrasion resistance strongly depends on the size of the denticles. Due to the dense tissue structure and calcification, the abrasion resistance increases as the denticle size increases, reaching its maximum at the denticle surface. The denticles of the mud crab have a tissue structure that prevents them from breaking when pinched. The high abrasion resistance of the large denticle surface is an essential feature for the frequent crushing of shellfish, which is the mud crab’s staple food. The characteristics and tissue structure of the claw denticles on the mud crab may provide ideas for developing stronger, tougher materials.

## 1. Introduction

The exoskeleton of crustaceans has a unique tissue structure that is not visible in man-made materials [1,2] and is superior in mechanical and functional properties [3,4]. Research on materials with excellent and new properties is being actively advanced by mimicking these structures [5,6,7,8,9,10].

**Figure 1:**
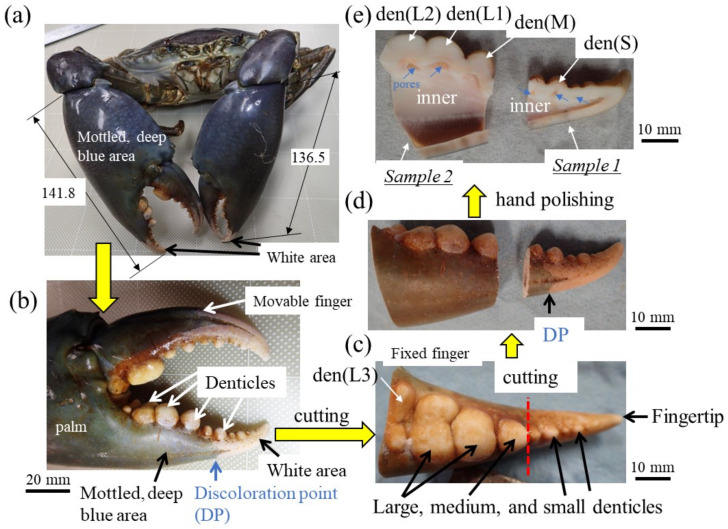
(**a**) The mud crab and (**b**) its right claw used in the present study. Large, medium, and small denticles lined up on the claw finger and the procedure before automatic polishing: (**c**) the fixed finger, (**d**) samples 1 and 2, and (**e**) cross-sectional photos Here, den(S) and den(M) denote small and medium denticles and den(L1), den(L2), and den(L3) denote large denticles.

Among crustaceans, the mud crab *Scylla serrata* has huge claws that exceed its body size [11,12]. The exoskeleton surface of the mud crab is mottled deep blue, while the claw top’s finger surface and the denticles are white (Figure 1a,b). In the mottled, deep-blue exoskeleton, the hard exocuticle layer (exoC) was 1.5–3.0% of the exoskeleton thickness, and the exoskeleton was dominated by the endocuticle layer (endoC) with a twisted-plywood pattern structure (TPS) commonly found in crustacean exoskeletons [13]. The TPS is generally referred to as the Bouligand structure, which is found in many biological materials [1]. On the other hand, in the white exoskeleton at the fingertip, the tissue had a TPS throughout the thickness, but the rigid and dense exoC occupied more than half of the exoskeleton thickness, and the hardness changed gradually at the boundary between the exoC and endoC [14]. The proportion of the exoC in the white exoskeleton was much higher than that in the mottled, deep-blue exoskeleton and in other crustaceans, including the brown crab, *Cancer pagurus* [15], the Chinese mitten crab, *Eriocheir sinensis* [16], the Atlantic blue crab, *Callinectes sapidus*, the Mediterranean green crab, *Carcinus aestuarii* [17], the coconut crab, *Birgus latro* [18,19,20], and the American lobster, *Homarus americanus* [21]. When a mud crab pinches a relatively large prey (such as other crabs), the white part of the fingertip is used to pinch the hard body of the prey and crush it (Figure S1a in the Supplementary Materials). The white parts of the claws are also used to make a loud sound to intimidate enemies (Video S1 in the Supplementary Materials) and to protect the weaker area near the mouth that is not covered by the rigid exoskeleton (Figure S1b,c) [22]. In other words, the white parts play a special role in effectively utilizing the huge claws to enable the mud crab to survive in its habitat. On the other hand, when a mud crab pinches a small prey such as a shellfish, which is its staple food, the denticles on the pinching side are the first point of contact and where a large force acts (Figure S1d). Unlike the case with the exoskeleton of the carapace and the outer side of the claws, there are few studies examining the microstructures and mechanical properties of the denticles of the pinching side [11,23]. In particular, in the case of the mud crab, denticles on the claw finger are characterized by being small (small denticles) at the fingertip and becoming larger (medium and large denticles) closer to the palm, as shown in Figure 1b. The mud crab captures small prey (such as clams) by pinching them with the base of their claws and crushing them (Figure S1d). Since the role of the denticles is different from that of the mottled, deep-blue exoskeleton and the white part of the fingertip, the mechanical properties and tissue structure may also differ and depend on the size of the denticle. In this paper, the mechanical properties, tissue structure, and chemical components of small, medium, and large denticles are investigated, and the results are compared with those of the blue exoskeleton and the fingertip.

## 2. Materials and Methods

A large male mud crab (body weight 1265 g, carapace length 113.4 mm, carapace width 167.3 mm) with a rigid exoskeleton was obtained live from a local market in Naha, Okinawa, Japan. The crab was stored frozen at −18 °C to prevent natural decay processes and transported to our laboratory. The large right claw of 141.8 mm length, 66.5 mm width, and 39.5 mm thickness was removed (Figure 1a,b), and a sample was cut from the fixed finger with a handsaw (Figure 1c). The fixed finger was larger than the maximum mounting cup we have, so it was cut in two (samples 1 and 2) with a handsaw (Figure 1d) and hand polished until the cross sections of all denticles were exposed (Figure 1e). After the samples were dried for 48 h or more, the mounting cup in which the samples were set was filled with epoxy and left to cure at 23 °C for 12 h. To ensure penetration of the epoxy into the sample voids, the samples were placed under a vacuum for 600 s soon after the epoxy was added. After that, each sample was ground with SiC papers, polished with a 9, 3, and 1 μm diamond suspension, and finally polished with a 0.05 μm alumina suspension. The cross-sectional images of samples 1 and 2 were made by merging multiple optical micrograph images. Before observation using a scanning electron microscope (SEM), Vickers hardness and nanoindentation tests were performed.

The Vickers and nanoindentation tests were conducted using a Shimadzu Micro Vickers Hardness Tester, HMV-G31 (SHIMADZU, Kyoto, Japan), and an ENT-NEXUS (ELIONIX, Tokyo, Japan). The Vickers measurement was taken with the application of 98.07 mN for 15 s. Nanoindentation testing was conducted with a Berkovich diamond indenter with an angle of 115°. The loading curve consisted of a 5 s loading to 5 mN, followed by a 5 s hold at that force, and then a 5 s unloading. The tests were performed at an interval of 50 μm from an outer surface to an inner surface. The hardness (*H_IT_*) and reduced elastic modulus (*E_r_*) were analyzed from the unloading curve using the Oliver–Pharr method [24] employed in biological studies [14,15,19,20,21].

To characterize the microstructure and chemical compositions, two samples were coated with about 2 nm of osmium (Neo Osmium Coater, Meiwafosis Co., Ltd., Tokyo, Japan) before SEM observation. A focused ion beam (FIB)–SEM dual-beam instrument (Scios2, Thermo Fisher Scientific, Waltham, MA, USA) at an accelerating voltage (AC) of 2 kV and a secondary electron detector in a chamber or an in-lens annular back-scattered electron detector was used for the microstructure characterization. An energy-dispersive X-ray spectroscope (EDS) attached to this FIB–SEM instrument was applied for the compositional analysis. A large silicon-drift detector (Ultim Max 170, Oxford Instruments, Abingdon, Oxfordshire, UK) ensured high detecting efficiency and low statistical error in the quantitative analysis. The EDS analysis was used at an AC of 15 kV. Due to the influence of the final alumina suspension in the polishing process, Al remained on the sample surface, so Al was excluded from the EDS quantitative analysis.

## 3. Results and Discussion

### 3.1. Mechanical Properties

**Figure 2:**
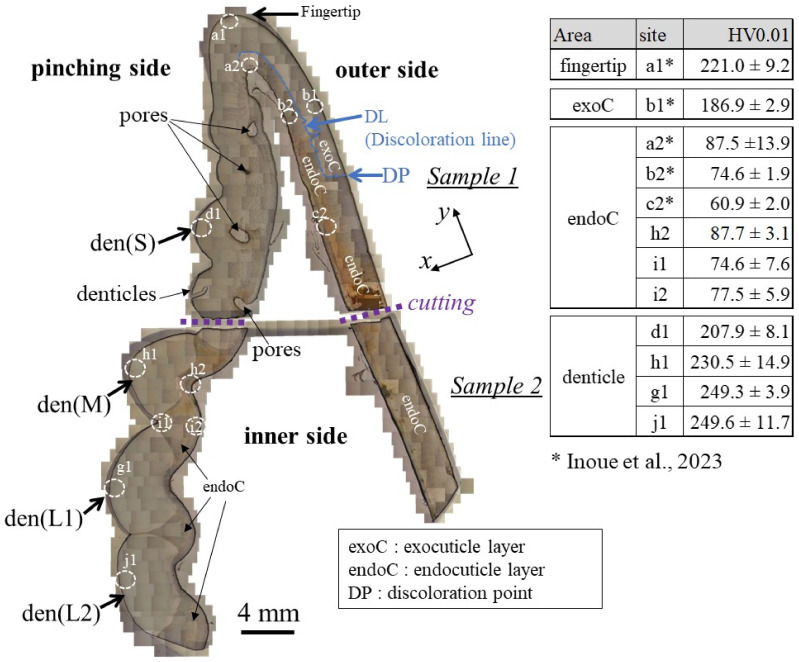
Optical micrographs of a cross-section of the entire fixed finger of the claw after polishing and the hardness at four characteristic areas were obtained via Vickers tests. The tests were performed more than five times for each site, and the site is 100–200 μm away from the outer and inner surfaces. Here, den(S) and den(M) denote small and medium denticles, and den(L1) and den(L2) denote large denticles [14].

The cross-sections of samples 1 and 2 are shown in Figure 2. The areas of the mineralized claw exoskeleton are classified as the fingertip, exoC, endoC, and denticle. Pores are visible in the exoskeleton with the denticles in sample 1. In sample 2, which has a large denticle, these pores appear as constrictions in the exoskeleton. The pores just below the denticle have also been observed in the coconut crab exoskeleton [20,23]. The results of the Vickers hardness test at characteristic sites for these four areas are represented in the table in Figure 2. Here, the Vickers hardness, HV0.01, is the average value, including the standard deviation. The HV0.01 of the endoC of the pinching side (site h2, i1, and i2) and of the fingertip (site a2) is almost the same as that of the outer side (site b2 and c2). On the other hand, the denticle surfaces were harder than the exoC of the outer side (site b1) and the endoC. The medium denticle (site h1) and large denticles (site g1 and j1) were harder than the fingertip (site a1). The HV0.01 increased with the increase in denticle size. Namely, the hardness depended on the size of the denticle. The value, 250HV0.01, for the two large denticles is comparable to that for the exoC of the coconut crab claw [18] and the maximum value for the exoC of the American lobster claw [21].

**Figure 3:**
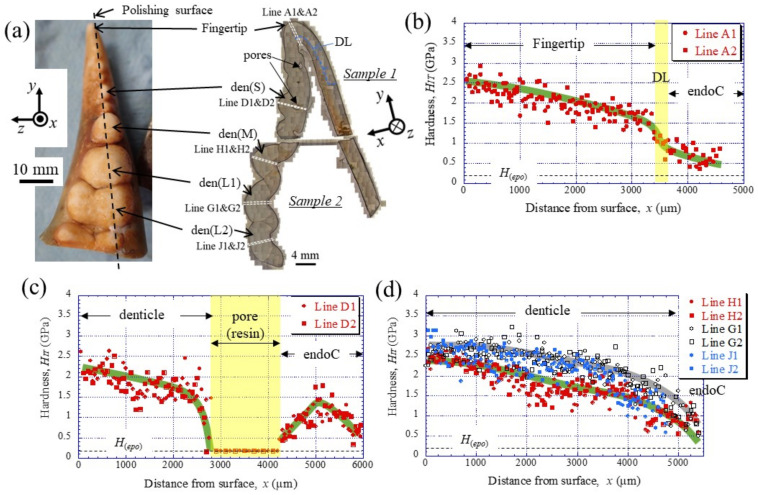
(**a**) Appearance of the fixed finger after cutting and cross-sectional micrographs after polishing. Distributions of hardness, *H_IT_*, with distance from the outer surface, *x*, on (**b**) Line A (fingertip), (**c**) Line D (small denticle), and (**d**) Line H (medium denticle) and Lines G and J (large denticles) in the claw cross-section, obtained via nanoindentation tests. Here, *H*_(*epo*)_ denotes the hardness of the cold epoxy resin, DL denotes the discoloration line, and endoC denotes the endocuticle.

**Figure 4:**
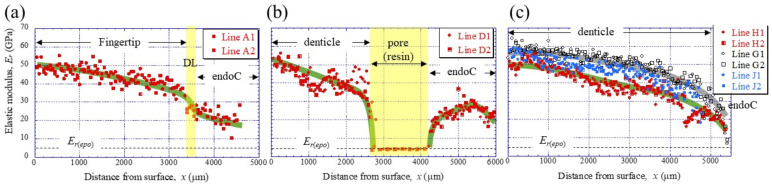
Distributions of the reduced elastic modulus, *E_r_*, with *x*, on (**a**) Line A (fingertip), (**b**) Line D (small denticle), and (**c**) Line H (medium denticle) and Lines G and J (large denticles) in the claw cross-section, obtained using nanoindentation tests. Each line is shown in Figure 3a. Here, *E*_*r*(*epo*)_ denotes the elastic modulus of the cold epoxy resin, DL denotes the discoloration line, and endoC denotes endocuticle.

To clarify the denticle size dependence on hardness, the change in mechanical properties, *H_IT_* and *E_r_*, along the thickness from the outer surface to the inner surface on the pinching side, was investigated through nanoindentation testing. Figure 3c,d shows the distribution of *H_IT_* along lines D1, D2 (small denticle), lines H1, H2 (medium denticle), and lines G1, G2 and J1, J2 (large denticles). For comparison, the results for lines A1 and A2 of the fingertip are shown in Figure 3b. Here, the two lines are parallel and separated by more than 50 μm at each thickness. In the fingertip (Figure 3b), the *H_IT_* gradually decreased from 2.5 GPa, decreased abruptly at the discoloration line (DL), and then gradually decreased again. The *H_IT_* in the small denticle (Figure 3c) decreased gradually from 2–2.5 GPa and then decreased abruptly as it approached the pore. In the medium and large denticles (Figure 3d), the *H_IT_* gradually decreased until the inner surface, like the changes, until the DL of the fingertip. The *H_IT_* of the two large denticles (lines G1, G2, and J1, J2) was almost the same and larger than that of the medium denticle (lines H1, H2). The results for the *E_r_*, which showed changes similar to those for the *H_IT_*, are summarized in Figure 4.

**Figure 5:**
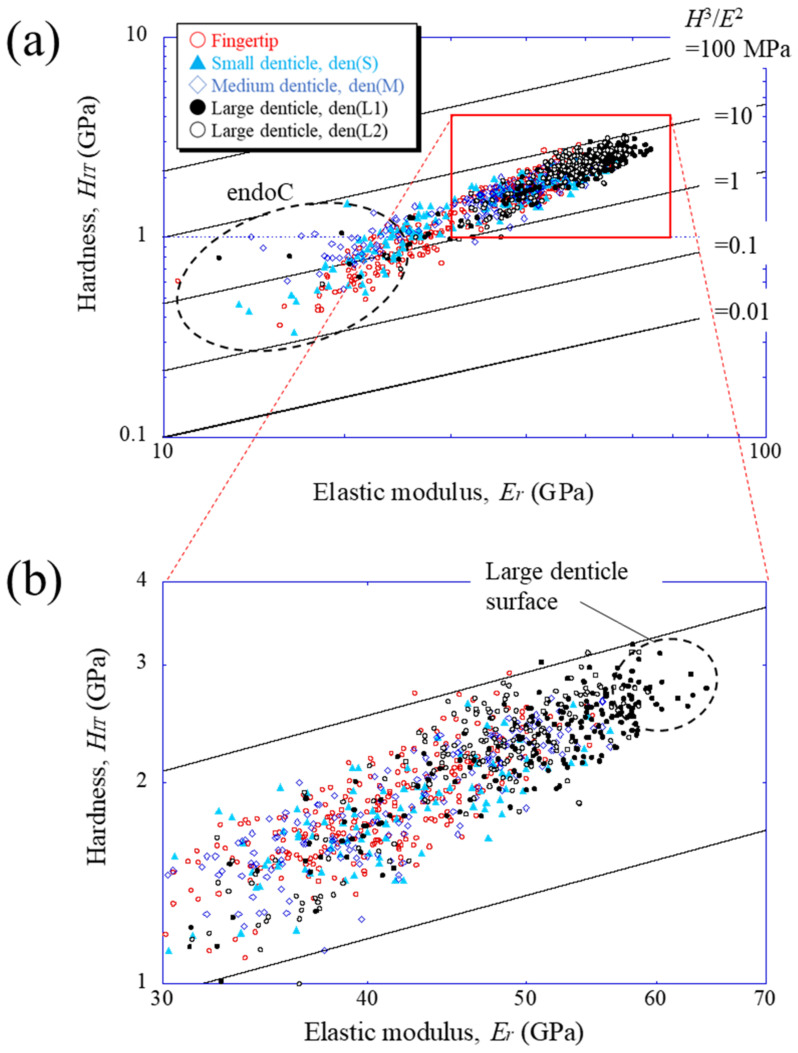
(**a**) Property map for abrasion resistance on Line A (fingertip), Line D (small denticle), Line H (medium denticle), and Line G and J (large denticles), shown in Figure 3a, and (**b**) enlarged map. Here, data lying on a straight line of *H*^3^/*E*^2^ indicate materials with equivalent performances in abrasion resistance.

To compare the mechanical properties of each denticle, the results were plotted on a map based on the abrasion resistance [25] of materials. Figure 5 shows the property maps with data for the fingertip and all denticles. The abrasion resistance near the two large denticle surfaces is higher than that in other areas. This means that the two large denticles closer to the palm among the denticles arranged in a line on the claw finger are mainly used when the mud crab pinches and eats small prey, as shown in Figure S1d in the Supplementary Materials. These values, 2.3 < *H_IT_* < 3.2 GPa and 56 < *E_r_* < 65 GPa, are the limits of the local mechanical properties of the fixed finger of the mud crab’s claw, and the abrasion resistance is classified into materials with 3.3 < *H*^3^/*E*^2^< 9.0. This is higher than that in the data of all engineering polymers and is comparable to data of the hardest metallic alloys and even some of the softer ceramics [19,25]. The map reveals that the *H_IT_*-*E_r_* balance decreases from the outer surface to the inner side and approaches the balance of the endocuticle layer.

### 3.2. Microstructure

**Figure 6:**
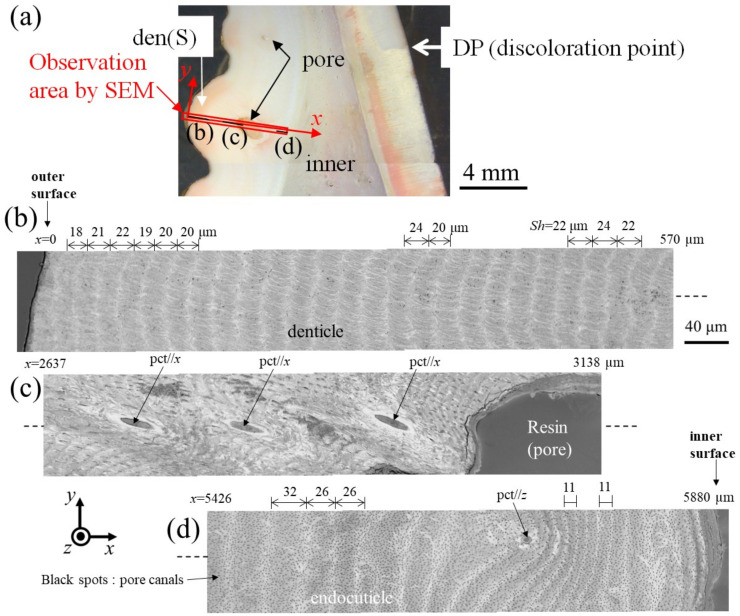
(**a**) Stereomicrograph of the claw cross section (sample 1). SEM micrographs of Line D, including the small denticle (den(S)): (**b**) *x* = 0–570 μm, (**c**) *x* = 2637–3138 μm, and (**d**) *x* = 5426–5880 μm. Here, pct//*x* and pct//*z* denote pore canal tubules parallel to *x* and perpendicular to the *x*-*y* plane, and *Sh* denotes the stacking height of the twisted-plywood pattern structure.

**Figure 7:**
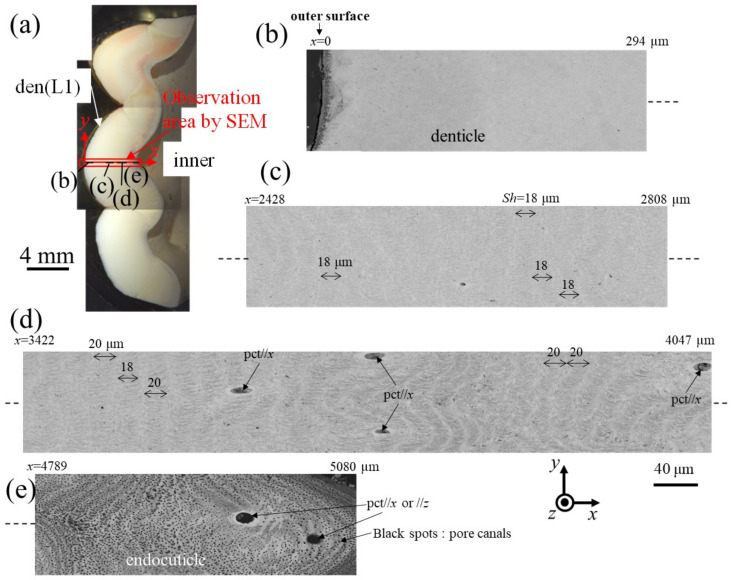
(**a**) Stereomicrograph of the claw cross section (sample 2). SEM micrographs of Line G, including the large denticle (den(L1)): (**b**) *x* = 0–294 μm, (**c**) *x* = 2428–2808 μm, (**d**) *x* = 3422–4047 μm, and (**e**) *x* = 4789–5080 μm. Here, pct//*x* and pct//*z* denote pore canal tubules parallel to *x* and perpendicular to the *x*–*y* plane, and *Sh* denotes the stacking height of the twisted-plywood pattern structure.

Figure 6 and Figure 7 show SEM micrographs near the outer surface, middle, and inner surface on Line D (small denticle, den(S)) and Line G (large denticle, den(L1)), shown in Figure 2. On Line D in Figure 6, the whole part of the exoskeleton with a 5880 μm thickness is striated parallel to the stacking height (*Sh*) of the TPS, and the *Sh* gradually decreases as it approaches the outer and inner surfaces. In particular, the *Sh* near the inner surface is very low, 11 μm or less. The *Sh* near the outer surface is 18–24 μm. Some pore-canal tubes (pcts)//*x* perpendicular to the outer surface were clearly observed near the pore filled with resin. The black spots in the endoC near the inner surface indicate pore canals (pcs). These black spots were also observed on the endoC on the outer side of the claw finger in the previous paper [14] and often can be observed on the polished surface of the exoskeleton with a TPS in crustaceans [1,18,20,23,26]. On the other hand, in Line G, shown in Figure 7, since the large denticle surface had a dense tissue structure, it was not possible to clearly observe the microstructure. The microstructures are visible from the middle to the inner surface and were striated with the *Sh* (18–20 µm) of the TPS, similar to Line D. Furthermore, many black spots were observed at the endoC near the inner surface.

### 3.3. Chemical Compositions

The EDS results for seven characteristic sites are summarized in Table 1. Calcium (Ca), magnesium (Mg), phosphorus (P), carbon (C), oxygen (O), and sodium (Na) were found to be the main components, and chloride and sulfur were present in minor amounts. This result was the same as that for the exoC in the mottled, deep-blue exoskeleton of the mud crab in the previous paper [13,14]. Here, the crystalline structures of the exoskeleton of the mud crab were calcite [14]. The minor components are found in the exoskeleton of the lobster, edible crab, and coconut crab [19,23,27]. The area scan results for inorganic matter (Ca, Mg, and P) for each site revealed differences in the compositions in the denticle, fingertip, and endoC. Curiously, the Na concentrations were almost identical within the exoskeleton, ranging from 0.7–1.0%. This amount is consistent with the result for the carapace of *S. paramamosain* [28], which belongs to the same genus. On the other hand, the Ca concentrations at site a1 on the fingertip and sites d1, g1, and g2 in the denticles are higher than those in the endoC (sites a2, d2, and g3), but the Mg concentrations are lower, and the P concentrations are zero. This trend is consistent with the results of Waugh et al. [11], which showed that the denticles contained less P than the surrounding cuticle of the mud crab’s claw and that the amount of MgCO_3_ present in the calcite was lower in the denticles than in the claw and carapace. The Ca concentration at site g1 of the large denticle surface is larger than that at site d1 of the small denticle surface and site a1 of the fingertip. In the large denticle, the Ca concentration decreases as it approaches the inner side. This reflects the gradual change in mechanical properties from the outer surface to the inner surface, as shown in Figure 3c,d and Figure 4c,d.

### 3.4. Denticles Arranged in a Line on the Claw Finger

The denticles have the same TPS as the endoC [13,14] and are described as a bulge in the endoC. For crustaceans, the denticles on the pinching side exist for the specialized function of pinching and crushing prey like teeth, as compared to the outer exoskeleton that exists to protect the body. Hence, it is necessary for the denticles to have high abrasion resistance and to disperse the force applied to the claw when pinching. In the mud crab, the denticles are arranged in a line on the fixed fingers of the claws, and their sizes become larger closer to the palm (Figure 1). This feature was common to all mud crabs sold at the local market in Naha and to those reported in the literature [11]. The mechanical properties increased with the increase in denticle size and reached their maximum at the denticle surface (Figure 3, Figure 4 and Figure 5). This was due to the dense tissue structure and calcification.

The mud crab inhabits the sandy bottom of relatively shallow inner bays [12,13,22]. The claw with denticles arranged in a line on the finger is found in the blue crab, *Callinectes sapidus* [11,17], and the green crab, *Carcinus maenas* [17], of the same Portunidae family and the land crab, *Discoplax hirtipes*, living on similar sandy bottoms, but not in terrestrial coconut crabs [23]. In the coconut crab, there are many large and small denticles irregularly on the pinching side of the claw, and relatively large denticles are arranged in a line on the front side, with very small denticles randomly arranged on the back side. The difference in these denticles on the claw finger should be associated with biomineralization in the crab’s habitat.

The mud crab, *Scylla serrata*, is omnivorous but consumes mainly shellfish. The large denticle (den(L2)) is relatively flat and wide compared to the small (den(S)) and medium denticles (den(M)), as shown in Figure 1c. When observed in detail, the top of den(L2) is slightly concave, and the adjacent denticle (den(L3)), opposite to den(L1), is split in two in the middle of its width. When a shellfish is placed on den(L2), it is sandwiched and stable between the convex part of den(L1) and the valley of den(L3). The fixed shellfish is crushed by a larger denticle on the pinching side of the movable finger, and the crabs can consume the contents of the shellfish. The high abrasion resistance of the large denticle surface shown in Figure 5 is an essential feature for the frequent crushing of shellfish. On the other hand, the fingertip of the same white exoskeleton is used to pinch large prey that cannot be captured and crushed by the denticles of the pinching side and to intimidate predators (Figure S1a,b). The curvature of the fingertips toward the pinching side allows the fingertips to pierce prey when pinched. Therefore, the fingertip is as hard as the large denticle surface.

### 3.5. Twisted–Plywood Structure Stacked Parallel to the Surface in the Denticles

**Figure 8:**
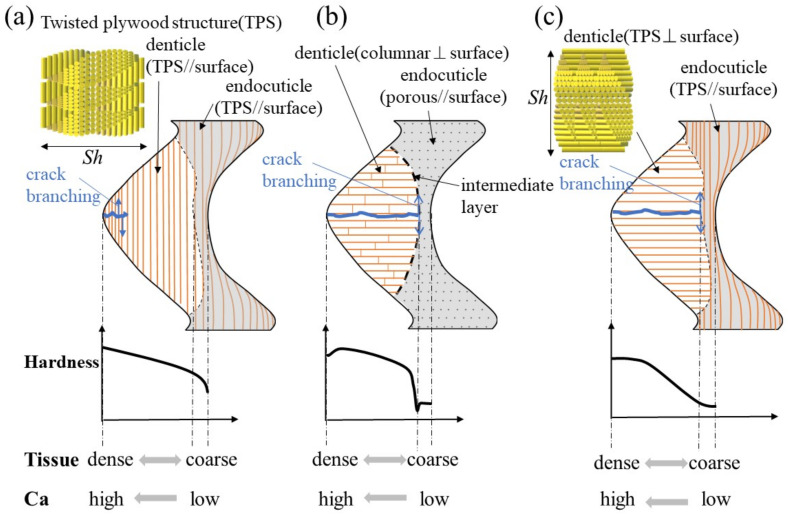
Schematic illustration of the exoskeleton tissue with denticle in the pinching side of claws and variations of hardness, tissue density, and calcium (Ca): (**a**) the mud crab, (**b**) the coconut crab [13,20], and (**c**) the brachyuran crab [26].

Figure 8 shows a schematic illustration of the exoskeleton tissue of the pinching side in three kinds of crab claws. The mud crab’s white exoskeleton (denticle) tissue is a tough TPS stacked parallel to the surface, and mechanical properties gradually decreased from the outer surface to the inner surface, as shown in Figure 8a. Rosen et al. [26] (two anomuran crabs, *Paralithodes camtschaticus* and *Paralomis birsteini,* and three brachyuran crabs, *Chionoecetes opilio*, *Callinectes sapidus*, and *Cancer borealis*) and Inoue et al. [23] (coconut crabs) have investigated the denticle microstructure in detail. Rosen et al. showed that the TPSs run parallel to the exoskeleton surface within the endoC but rotate ~90° (perpendicular to the exoskeleton surface) as they approach the denticle region (Figure 8c). That is, the denticles of the crabs have a TPS stacked perpendicular to the surface. Inoue et al. showed that the denticle in the coconut crab was a columnar structure perpendicular to the surface (Figure 8b). The denticle tissue of all these crustaceans runs perpendicular to the surface, although the tissue patterns are different. With this tissue structure, cracks tend to advance inside, and the claws are more likely to be damaged. However, since the intermediate layer between the denticle and the endoC is soft, only the denticles are considered to be lost or damaged before the claws are fractured. If some denticles are lost, the other denticles maintain the function of the claws, and then new denticles develop by molting.

On the other hand, for the mud crab, losing a denticle is directly related to not being able to crush a staple food. Therefore, in the exoskeleton of the pinching side with large denticles, there was almost no endoC (Figure 2), and no rapid decrease in hardness was observed in the intermediate layer (Figure 3b–d). In addition, since the structure of the denticle was a twisted–plywood pattern stacked parallel to the surface, it was tough, making it difficult for cracks to propagate inside, as shown in Figure 8a. In short, the denticles of the mud crab have a tissue structure that is never lost if they crack while pinching. Such a structure has also been observed in the tissue structure of hypermineralized hammer-like dactyl clubs, a weapon for crushing prey [29].

## Figures and Tables

**Table 1:** EDS area scan results at seven areas of the samples. Here, the results show the average weight % of calcium (Ca), magnesium (Mg), phosphorus (P), carbon (C), oxygen (O), sodium (Na), chlorine (Cl), and sulfur (S).

Area	Site	Ca (wt%)	Mg (wt%)	P (wt%)	C (wt%)	O (wt%)	Na (wt%)	Cl (wt%)	S (wt%)
fingertip	a1	30.3	0.9	0.0	16.2	51.7	0.9	0	0.0
endocuticle	a2	24.9	2.2	0.7	20.9	50.4	0.8	0.1	0.1
denticle	d1	29.5	1.1	0.0	16.6	51.7	0.9	0.1	0.1
endocuticle	d2	24.7	2.1	0.2	22.3	49.7	0.8	0.2	0.1
denticle	g1	31.1	1.3	0.0	14.3	52.3	1.0	0.0	0.0
denticle	g2	29.8	1.4	0.0	15.6	52.1	1.0	0.1	0.0
endocuticle	g3	25.5	2.3	0.6	22.1	48.6	0.7	0.2	0.1

## Data Availability

Not applicable.

## Supplementary Materials

The following supporting information can be downloaded at: https://www.mdpi.com/article/10.3390/ma16114114/s1: Figure S1: Schematic illustrations of predation, attack, and defense of the mud crab, *Scylla serrata*, with huge claws comparable to its body size; Video S1: The mud crab’s intimidation.
