# Supplementary material for: Mechanical Resistance and Tissue Structure of Claw Denticles of Various Sizes in the Mud Crab, Scylla serrata
Source: Materials (Basel). 2023 May 31;16(11):4114. doi: 10.3390/ma16114114 (PMC10254728; doi:10.3390/ma16114114)
Supplement: Supplementary file 1 [file materials-16-04114-s001.zip › Figure S1.pdf]

Supplementary Materials

Mechanical Resistance and Tissue Structure of Claw Denticles of Various Sizes in the Mud Club, *Scylla serrata*

Tadanobu Inoue, Yuka Hara and Koji Nakazato

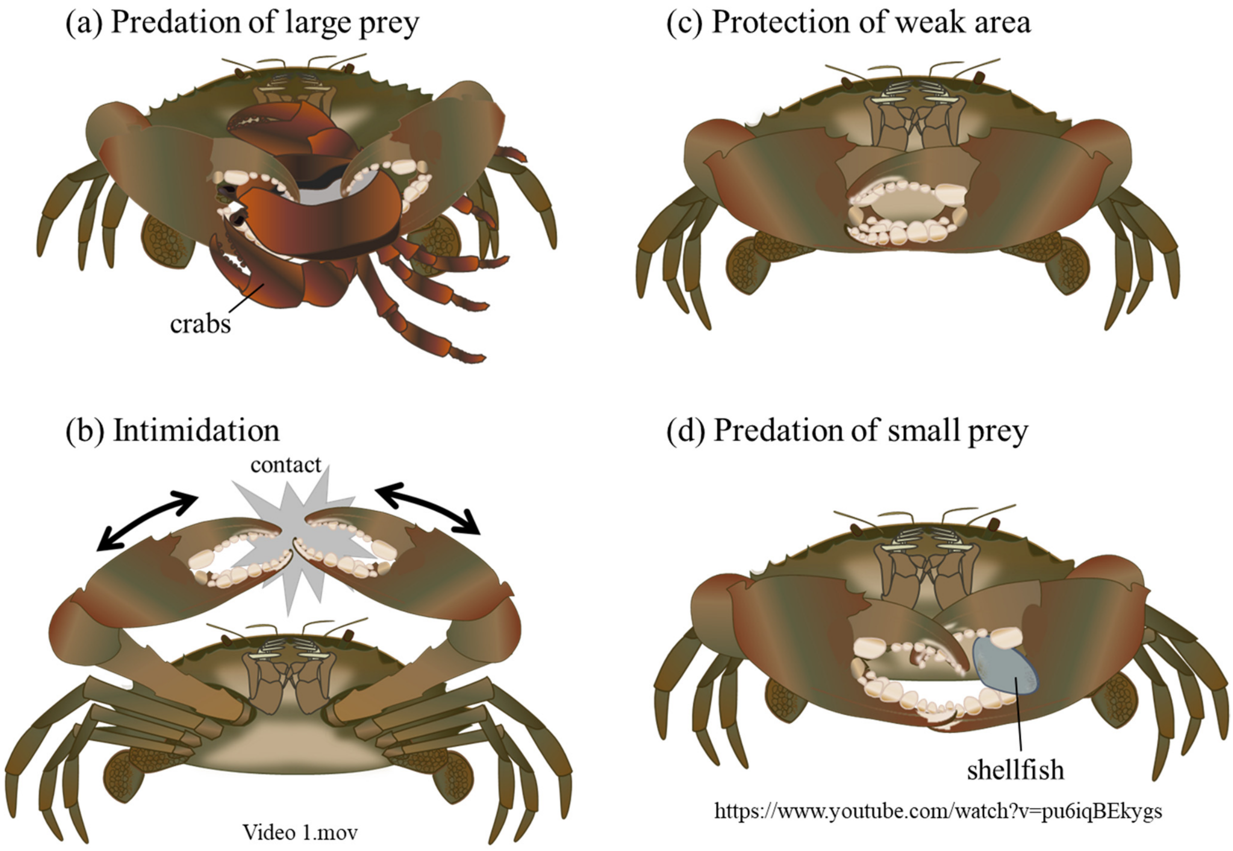

Figure S1. Schematic illustrations of predation, attack, and defense of the mud crab, *Scylla serrata*, which has huge claws comparable to its body size.

Video S1.mov. The mud crab's intimidation.
